# Rheocarna Effectively Treats Oral Medicinal Therapy‐Resistant Cutaneous Symptoms and Renal Failure Induced by Cholesterol Crystal Embolism

**DOI:** 10.1002/ccr3.9608

**Published:** 2024-11-20

**Authors:** Akinori Satake, Takahiro Tokuda, Hirofumi Ohashi, Yusuke Nakano, Akio Kodama, Tetsuya Amano

**Affiliations:** ^1^ Department of Cardiology Narita Memorial Hospital Toyohashi Aichi Japan; ^2^ Department of Cardiology Nagoya Heart Center Nagoya Aichi Japan; ^3^ Department of Cardiology Aichi Medical University Nagakute Aichi Japan; ^4^ Department of Vascular Surgery Aichi Medical University Nagakute Aichi Japan

**Keywords:** cholesterol crystal embolism, cutaneous symptoms, low‐density lipoprotein apheresis, renal failure, Rheocarna

## Abstract

An 80‐year‐old man presented to our hospital with worsening renal function and ambulation difficulties due to lower extremity symptoms that included livedo reticularis, gangrene, cyanosis, and ulcers in his legs. The patient was diagnosed with a cholesterol crystal embolism. Treatment with prednisolone and rosuvastatin was initiated; however, no improvements were observed in the patient's cutaneous symptoms or renal function. Therefore, we decided to treat the patient with a new low‐density lipoprotein apheresis device (Rheocarna; Kaneka Corporation, Osaka, Japan). Following therapy, the lower extremity symptoms improved, and although dialysis was temporarily initiated, it was eventually discontinued.

**Summary**

- Currently, no definitive treatment exists for cholesterol crystal embolism (CCE); however, low‐density lipoprotein apheresis (LDL‐A) has been reported as a treatment.
- A new LDL‐A device, Rheocarna (Kaneka Corporation, Osaka, Japan), may offer an effective approach for managing CCE.

## 1 Introduction

Cholesterol crystal embolism (CCE) occurs when cholesterol crystals embolize from the proximal artery to the small distal arteries, resulting in end‐organ damage due to mechanical plugging and an inflammatory response. CCE can lead to renal failure and skin damage [1].

Earlier studies revealed that statins and steroids are effective in the treatment of CCE [2], although no definitively established treatment currently exists. Low‐density lipoprotein apheresis (LDL‐A) has been reported to treat CCE [3]. Recently, a new LDL‐A device (Rheocarna; Kaneka Corporation, Osaka, Japan) was developed for chronic limb‐threatening ischemia with refractory ulcers and enabled clinicians to remove low‐density lipoprotein cholesterol (LDL‐C) and fibrinogen [4, 5].

Notably, little is known about the effectiveness of Rheocarna in treating CCE. We successfully used Rheocarna to treat a patient with CCE who had cutaneous symptoms and renal failure that did not respond to oral medications.

## 2 Case Presentation

**FIGURE 1:**
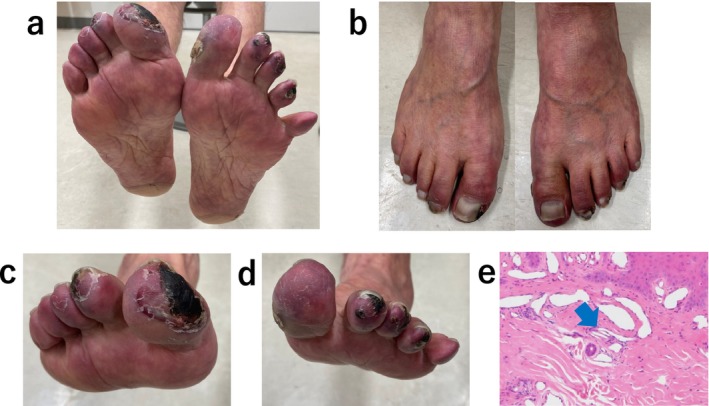
(a–d) Wound appearance at admission. (e) A cutaneous biopsy specimen showing needle‐shaped cholesterol cleft gaps within the arterioles (green arrow).

An 80‐year‐old man presented to our hospital with worsening renal function and ambulation difficulties due to lower extremity symptoms. Two months prior, he had undergone endovascular aortic repair and bilateral renal artery stent implantation to treat an abdominal aortic aneurysm at another hospital. His medical history included smoking, hypertension, dyslipidemia, and chronic kidney disease. His medications included bisoprolol (5 mg/d), aspirin (100 mg/d), vonoprazan (10 mg/d), and nifedipine (20 mg/d). His physical examination revealed livedo reticularis, gangrene, cyanosis, and ulcers on his legs (Figure 1a–d).

### 2.1 Investigations and Management

**FIGURE 2:**
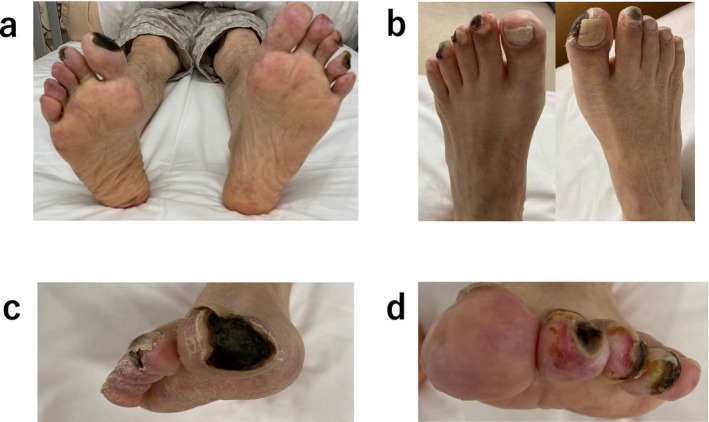
(a–d) Wound appearance after 24 sessions of the first‐course Rheocarna therapy.

**FIGURE 3:**
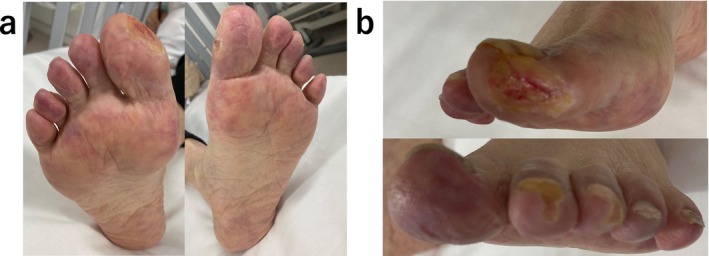
(a, b) Wound appearance before the second‐course Rheocarna therapy.

**FIGURE 4:**
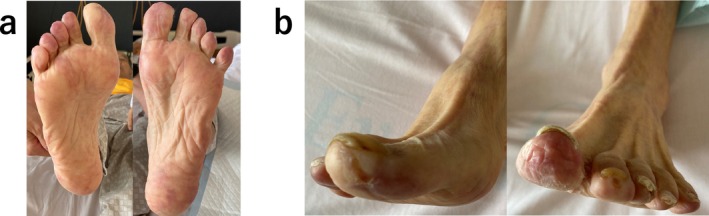
(a, b) Wound appearance after the second‐course Rheocarna therapy.

Electrocardiography at admission revealed a normal sinus rhythm. Blood analysis showed creatinine levels of 3.48 mg/dL and an estimated Glomerular Filtration Rate (eGFR) of 14 mL/min/1.73m^2^; urine protein was positive. Six months prior to this visit, creatinine levels were 1.91 mg/dL, and the eGFR was 27 mL/min/1.73 m^2^. A skin biopsy was performed, diagnosing the patient with CCE based on the needle‐shaped cholesterol cleft gaps evident on histological examination (Figure 1e, blue arrow). Treatment was initiated with prednisolone (30 mg/day) and rosuvastatin (2.5 mg/day); however, no improvement was observed in the patient's cutaneous symptoms or renal function. Oliguria gradually progressed, and despite continuing medical treatment, hemodialysis was initiated using a catheter inserted into the right internal jugular vein 3 weeks after the patient's visit. Hemodialysis was performed for 2 weeks without improvement in renal function, leading to the creation of an arteriovenous fistula. Prednisolone (30 mg/day) and rosuvastatin (2.5 mg/day) were administered for 4 months, but there was no improvement in the cutaneous symptoms. Subsequently, we began Rheocarna therapy using the fistula; the LDL‐C, fibrinogen, and C‐reactive protein (CRP) levels before Rheocarna therapy were 52, 322, and 0.68 mg/dL, respectively. Rheocarna was used for 24 sessions (maximum) over 2 months; blood flow was maintained at 100–120 mL/min for 2 h each session, three times a week on non‐dialysis days. Apheresis was performed through the arteriovenous fistula used for hemodialysis. Nafamostat was used as an anticoagulant; no vasodilators were used during the therapy. No complications occurred with the Rheocarna therapy; although the gangrene and ulcers did not completely heal, the livedo reticularis and cyanosis improved dramatically (Figure 2a–d). The patient regained enough strength to walk. His LDL‐C levels decreased from 52 to 32 mg/dL, fibrinogen from 322 to 197 mg/dL, and CRP from 0.68 to 0.08 mg/dL. However, the dorsal and plantar skin perfusion pressures (SPPs) were almost unchanged (Table 1). The status of the leg gradually improved; however, the ulcer on the first toe of the right leg did not heal completely (Figure 3a,b). Two months after the initial therapy, Rheocarna therapy was restarted. The patient's LDL‐C, fibrinogen, and CRP levels before the second Rheocarna therapy were 81, 236, and 0.24 mg/dL, respectively. His dorsal and plantar SPPs were near‐completely maintained, compared to those after 24 sessions of Rheocarna therapy (Table 1). After the second set of 24 sessions of Rheocarna therapy, the ulcer on the first toe of the right leg completely healed (Figure 4a,b), and the patient's LDL‐C levels decreased from 81 to 21 mg/dL. However, his fibrinogen and CRP levels increased from 236 to 306 mg/dL and 0.24 to 2.82 mg/dL, respectively. The dorsal and plantar SPPs were maintained (Table 1). Two cycles of Rheocarna completely healed the ulcers. Ten days after completing the second round of Rheocarna, the patient's dialysis was discontinued, and his creatinine levels improved (Table 2).

**TABLE 1:** The clinical course of SPP, LDL‐C, fibrinogen, and CRP levels.

	Before Rheocarna for the first course	After 24 sessions of Rheocarna for the first course	Before Rheocarna for the second course	After 24 sessions of Rheocarna for the second course
Skin perfusion pressure in dorsal (right/left)	89/63	72/57	85/69	75/87
Skin perfusion pressure in plantar (right/left)	86/84	83/84	82/74	79/78
LDL‐C (mg/dL)	52	32	81	21
Fibrinogen (mg/dL)	322	197	236	306
CRP (mg/dL)	0.68	0.08	0.24	2.82

**TABLE 2:** The clinical course of creatinine levels.

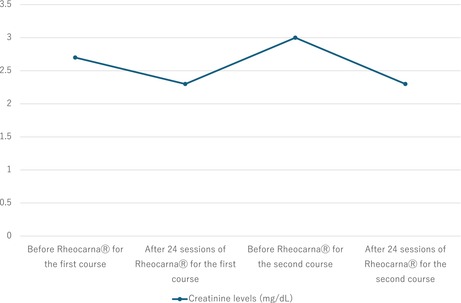

## 3 Results and Follow‐Up

The patient was discharged completely healed of ulcers and was regularly followed up at the outpatient clinic. The wound status was assessed at each visit, and there was no recurrence.

## 4 Discussion

To our knowledge, this is the first report assessing the effectiveness of Rheocarna for oral medicinal therapy‐resistant cutaneous symptoms and renal failure caused by CCE. Rheocarna, an adjuvant therapy, is a novel LDL‐A treatment that eliminates both LDL‐C and fibrinogen, unlike traditional LDL‐A (Liposorber; Kaneka Corporation, Osaka, Japan), which only target LDL‐C [6]. Rheocarna therapy may improve SPP, LDL‐C, fibrinogen, CRP levels, and microcirculation [7, 8]. SPP measurement is a reliable method for evaluating microcirculation and is not influenced by arterial calcification [9]. An SPP ≥ 40 mmHg accurately predicts wound healing [10, 11].

The patient's SPP was maintained above 40 mmHg on admission and throughout the treatment period, indicating that microcirculation was maintained. LDL‐A has been reported to improve endothelial function by reducing serum levels of total LDL and oxidized LDL and reducing circulating inflammatory cytokines and chemokines [12]. Previous studies have reported that fibrinogen apheresis may also improve endothelial function [13]. In this patient, the improvement of cutaneous symptoms and renal failure likely occurred through the improvement of endothelial function via the anti‐inflammatory effects of two cycles of Rheocarna. On the other hand, the improvement in renal failure was considered to be due to the improvement of microcirculation resulting from the use of Rheocarna [14].

While the precise mechanism behind the effects of Rheocarna remains unclear, Rheocarna usage may improve cutaneous symptoms and renal failure in patients with CCE. Additional prospective studies with larger and more diverse patient cohorts should be conducted to determine the generalizability of our findings.

## 5 Conclusion

Rheocarna may provide a viable treatment option for patients with skin manifestations and renal failure caused by cholesterol crystal embolism.

## Author Contributions

**Akinori Satake:** writing – original draft. **Takahiro Tokuda:** supervision. **Hirofumi Ohashi:** writing – review and editing. **Yusuke Nakano:** writing – review and editing. **Akio Kodama:** writing – review and editing. **Tetsuya Amano:** writing – review and editing.

## Consent

The patient gave written informed consent to the publication of the details of the medical case and accompanying images.

## Conflicts of Interest

The authors declare no conflicts of interest.

## Data Availability

Data sharing is not applicable to this article as no new data were created or analyzed in this study.
